# Training a cataract surgeon

**Published:** 2014

**Authors:** M Babar Qureshi, M Daud Khan

**Affiliations:** Senior Medical Advisor: CBM East Mediterranean Region, Cambridge, UK.; Chairman: Board Of Directors, CHEF International, Islamabad, Pakistan.

**Figure:**
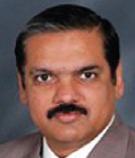
M Babar Qureshi

**Figure:**
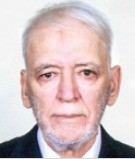
M Daud Khan

Training in cataract surgery is one of the key factors needed to ensure high quality cataract surgery with good visual outcomes and patient satisfaction. The training has to impart the right skills to the right person by the right trainer and in the right environment.

## Identification of tasks

Cataract surgery is now, in effect, refractive surgery, which involves more than just removing the opaque lens. It includes thorough pre-operative assessment, skilled surgical techniques and proper post-operative follow-up with a focus on the best possible recovery and visual outcome.

The tasks a cataract surgeon is expected to master include:

1. **Case selection *(Selection)***. The cataract surgeon should have thorough knowledge of the patients before surgery. Diseases such as corneal scars, age-related macular degeneration, diabetic retinopathy, advanced glaucoma and other comorbidities must be identified, because cataract surgery in their presence will not give the desired results.^1^ This has to be recorded and explained to the patient.
2. **Sterility and the surgical field *(Sterility)***. Procedures such as effective ‘scrubbing’, ‘gowning’ and ‘gloving’ should be strictly observed. Cleaning the peri-orbital skin with povidone iodine prior to surgery will reduce the bacterial load and can prevent post-operative endophthalmitis.^2^
3. **Anaesthesia and intraocular pressure *(Soft eye)***. A soft, well-anaesthetised eye is vital to the success of cataract surgery. Peribulbar injections and intermittent digital pressure are well suited for trainee surgeons and technicians. Note that sub-Tenon's also provides effective anaesthesia and has a lower incidence of sight-threatening complications compared with sharp needle techniques.^3^
4. **Availability and efficient use of suitable, sterilised instruments and optical aids/operating microscopes *(Suitable equipment)***. The candidate should have access to properly sterilised, good quality surgical instruments and have the use of a good quality and affordable microscope.
5. **Intra-operative surgical complications *(Safe surgery)***. The cataract surgeon should have good control over:
  - wound construction
  - capsulotomy
  - hydrodissection
  - nucleus delivery
  - cortex irrigation and aspiration
  - lens implantation
  - wound reconstruction.
  A safe cataract surgeon should know how to respect corneal endothelium, uveal tissues and the posterior capsule, and should avoid any damage to such tissues. In the case of posterior capsular rupture, he/she should know how to manage vitreous loss.
6. **Uncorrected refractive errors *(Spectacles)***. Significant astigmatism and uncorrected refractive errors from lost or broken glasses are an important cause of low vision and blindness following cataract surgery. It can be overcome in the following ways.
  - Biometry and the implantation of a customized intraocular lens (IOL) that will ensure significant improvement in visual outcome.
  - Small incision sutureless cataract surgery (SICS) or the appropriate removal of sutures to reduce significant astigmatism, followed by spectacle correction of the residual refractive error 6–8 weeks after surgery.^4^
7. **Post-operative complications *(Sequelae)***. There may be early or late complications. Persistent inflammation in the early post-operative period and posterior capsule opacification in the late period can adversely affect visual results. To avoid or minimise these, a cataract surgeon should take particular care in post-operative follow-up, with early detection and treatment of complications. Routine follow-up on the first post-operative day, after one week and after six weeks is recommended.^3^

**Figure:**
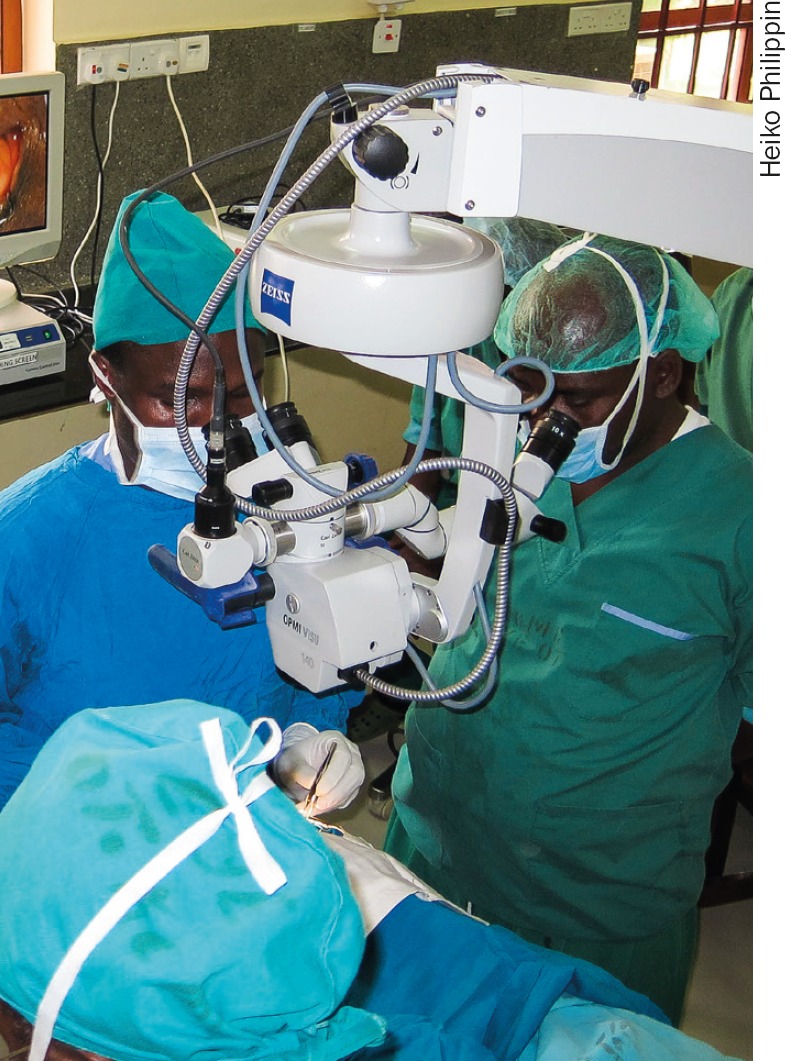
Trainers should be highly skilled and have an aptitude for teaching

## Training: length and content

Based on the above skills, a curriculum needs to be designed that gives the cataract surgeon the right knowledge, the right skills and the appropriate attitude. There will be considerable individual variations but, as a minimum standard, a person who is already qualified should receive 4–6 weeks of training in extracapsular cataract extraction (ECCE) with IOL and a minimum of fifty supervised operations is recommended.

Training should include didactic teaching, videos, wet lab and ‘hands-on’ training (assisted and then solo).

Training should be an ongoing process and not a one-time activity. Eye surgeons should get an opportunity to refresh their skills and learn new techniques.

Refresher training opportunities should be available according to the needs of the surgeons and should be offered as part of continuous professional development (CPD). CPD ensures not only that the eye surgeons continue to maintain their original set of skills, but also that they learn new skills with a view of continually updating themselves with the ever growing developments in cataract surgery.

## Auditing

Auditing or monitoring in the initial phase should allow eye surgeons to compare ‘themselves with themselves’ overtime. This can be done by measuring visual outcomes and patient satisfaction and should be an integral part of the training.

## Certification

Certification of training is the responsibility of the trainer who should be highly skilled and have an aptitude for teaching and training. The trainer is responsible for certifying trainees as safe cataract surgeons or recommending further training under supervision. Each trainee should have a log book which the trainer fills in and signs off at regular intervals and after the acquisition of the required skills.

## The training centre

Hospitals used as training centres for cataract surgery should have:

- adequate physical space
- adequate equipment and good quality instruments and consumables, as requested and required
- a ‘wet’ laboratory for the trainees to familiarise themselves with the instruments and microscope
- audio-visual systems for the recording of operations to enable learning, monitoring and for further reference.

**Tips for training in surgical skills**

The cornerstones of achieving good outcomes from cataract surgery are supervised training and practice. Skill and experience are necessary to achieve good outcomes. For these to be the norm the following are required:

- knowledge of the procedure
- supervised training
- practical surgical exposure and practice
- experience
- follow-up and audit of outcomes.

**Tips for the trainee**

- Watch and observe surgery and write down the steps in a notebook.
- Master the microscope and instruments.
- Scrub with the nursing team to appreciate and anticipate the steps involved.
- Break the procedure up into small sections.
- Practise, practise, practise!
- Practise in a wet lab, on plastic eyes or animal eyes and practise capsulorrhexis on a tomato or grape.
- Attend a microsurgical skills course.

**Tips for the trainer**

- Dedicate a set time on each list for the trainee, e.g. 40 minutes at the start of the list, after which time take over the case, whatever point has been reached. This time can be flexible depending upon the confidence of the trainer, competence of the trainee and the type of case.
- If the trainee needs practice in one particular step, supervise the trainee for that step in each case.
- ‘Reverse’ training is a method of learning the procedure from the end backwards e.g. suture tying first (in the case of ECCE).
- A positive attitude and approach is essential to encourage rather than humiliate the trainee.
- Discuss what went well and what didn't. Identify areas that need more practice.
- Frequent and regular exposure to surgery and to facilities for practice outside the theatres are essential.

Excellent outcomes can only be achieved through structured training, practising and personal audit of outcomes. Only then will patients truly benefit and the eye unit command respect in the community.

By Larry Benjamin, adapted by Nick Astbury. First published in the Community Eye Health Journal, Vol 15 No. 42, 2002.

## References

[1] Rao GN. Human Resource Development Comm Eye Health J. 2000;13:42–43 PMC1705979 17491961

[2] Kuriakose Thomas R Surgical techniques for a good outcome in cataract surgery: personal perspectives Comm Eye Health J. 2000;13:38–39 PMC1705975 17491959

[3] Guise P. Sub-Tenon's anesthesia: an update. Local Reg Anesth 2012;35–46 Available online: www.ncbi.nlm.nih.gov/pmc/articles/PMC3417980/10.2147/LRA.S16314 PMC3417980 22915900

[4] Cook C. How to improve the outcome of cataract surgery Comm Eye Health J. 2000;13:37–38 PMC1705972 17491958

